# The Death of George Fleming

**Published:** 1901-05

**Authors:** 


### THE DEATH OF GEORGE FLEMING.

WE again have the sad duty of publishing the obituary of one of the most distinguished veterinarians of the end of the last century. We reproduce from the *Veterinary Record*, April 27, 1901, the portrait and an abstract of the life-history and works of Mr. Fleming. While Mr. Fleming was so much better known to those in Great Britain, all veterinarians owe him a debt of gratitude for his books; and we in person and many Americans carry an affectionate remembrance of his genial hospitality and kind interest when we have called on him in London.

